# Quasi-BICs enhanced second harmonic generation from WSe_2_ monolayer

**DOI:** 10.1515/nanoph-2024-0108

**Published:** 2024-06-03

**Authors:** Peiwen Ren, Zhuo Huang, Song Luo, Jia Liu, Xiaoxiang Dong, Hua Zhang, Jianfeng Li, Zhilin Yang

**Affiliations:** College of Physical Science and Technology, State Key Laboratory of Physical Chemistry of Solid Surfaces, 12466 Xiamen University, Xiamen 361005, China; College of Chemistry and Chemical Engineering, State Key Laboratory of Physical Chemistry of Solid Surfaces, 12466 Xiamen University, Xiamen 361005, China

**Keywords:** bound states in the continuum, second-harmonic generation, 2D materials, WSe_2_

## Abstract

Quasi-bound states in the continuum (quasi-BICs) offer unique advantages in enhancing nonlinear optical processes and advancing the development of active optical devices. Here, the tunable robust quasi-BICs resonances are experimentally achieved through the engineering of multiple-hole Si-metasurface. Notably, the quasi-BICs mode exhibits flat bands with minimal dispersion at a wide range of incident angles, as demonstrated by the angle-resolved spectroscopy measurements. Furthermore, we demonstrate a giant second-harmonic generation (SHG) enhancement by coupling a WSe_2_ monolayer to the quasi-BICs hosted in the metasurface. Leveraging the strong local electric field and high state density of the observed quasi-BICs, the SHG from the WSe_2_ monolayer can be enhanced by more than two orders of magnitude. Our work paves the way for effectively enhancing nonlinear optical processes in two dimensional (2D) materials within the framework of silicon photonics and is expected to be applied in nonlinear optical devices.

## 1 Introduction

The development of novel on-chip nonlinear devices has put forward higher requirements for optical nonlinearity, which not only requires highly efficient and sensitive nonlinear conversion but also demands compatibility with current semiconductor techniques. Therefore, the enhancement of nonlinear processes is particularly important. Second-harmonic generation (SHG), as the lowest order optical nonlinearity that results from the second-order susceptibility tensor, has received much interest and has been extensively investigated in recent years [1], [2], [3]. Conventional SHG techniques rely on utilizing complex phase matching [4], and most nonlinear media are typically bulky crystals [5], [6], which limits their broad applicability, especially in terms of flexible on-chip integration. In the past two decades, two-dimensional transition metal dichalcogenides (TMDCs) have emerged as powerful candidates for constructing on-chip integrated nonlinear devices thanks to their ultrathin thicknesses and large nonlinear susceptibilities arising from the intrinsically broken crystal inversion symmetry [7], [8], [9], [10]. However, the atomic-scale-interaction-distance with light leads to the inherently weak nonlinearity of these materials [11].

As coupling with optical mode can further boost the photon state density or light field localization in materials, integrating nonlinear materials with resonant nanostructures can provide a promising way to enhance the efficiency of nonlinear processes [12], [13], [14]. This has been demonstrated in several representative cavity systems, including plasmonic cavities, distributed Bragg-reflector cavities, waveguides, and photonic crystals [15], [16], [17], [18]. Nevertheless, previously designed cavity structures either suffer from the relatively low state density in optical mode, or they are incompatible with on-chip integration due to their bulky nature, severely limiting their future practical applications. Bound states in the continuum (BICs) are highly localized states with infinite *Q*-factor (or lifetime), presenting great advantages for light–matter interactions [19], [20], [21]. Specifically, the use of localized modes is achievable via the production of high-quality quasi-BICs by manipulating the geometric parameters to control the radiative losses of BIC. This has been demonstrated in subwavelength-thick metasurfaces composed of dielectric meta-atoms in several recent studies [22], [23], [24], [25]. Typically, the excitation of BIC is constrained to collimated light at a small range of specific incident angles, and moreover, the resonance wavelength and quality factor of corresponding quasi-BIC are significantly influenced by changes in the incident angle [26]. Achieving quasi-BICs with flat bands is a key step to addressing these limitations and recent theoretical investigations have highlighted the prospective significance of BICs in fostering the emergence of photonic flat bands [27]. In this regard, all-dielectric metasurfaces with tunable quasi-BICs with flat bands present a promising alternative for complementing and even outperforming the capabilities of previously designed structures, as they hold the potential to provide a versatile and compact platform for both controlling optical modes and integration with two-dimensional materials for enhancing light–matter interactions and, consequently, the efficiency of nonlinear processes.

In this work, we engineer multiple-hole Si-metasurfaces and harness the quasi-BICs mode to enhance the SHG in a TMDs monolayer. By manipulating the tunable parameters of the presented metasurface, the quasi-BICs mode with a flat band is achieved, which is confirmed experimentally by angle-resolved spectroscopy. Remarkably, such quasi-BICs offer a vast enhancement in the state density and the local electric field in the optical mode, which makes perfect sense to boost the SHG in TMDCs materials. As a result, a giant enhancement of SHG emission from the TMDCs monolayer integrated on top of the metasurface is experimentally enhanced up to more than two orders of magnitude relative to the other regions of the same sample not located on the metasurface. This work offers an effective route towards silicon-chip-integrated nonlinear devices based on Si-metasurfaces combined with 2D materials.

## 2 Results and discussion

**Figure 1:**
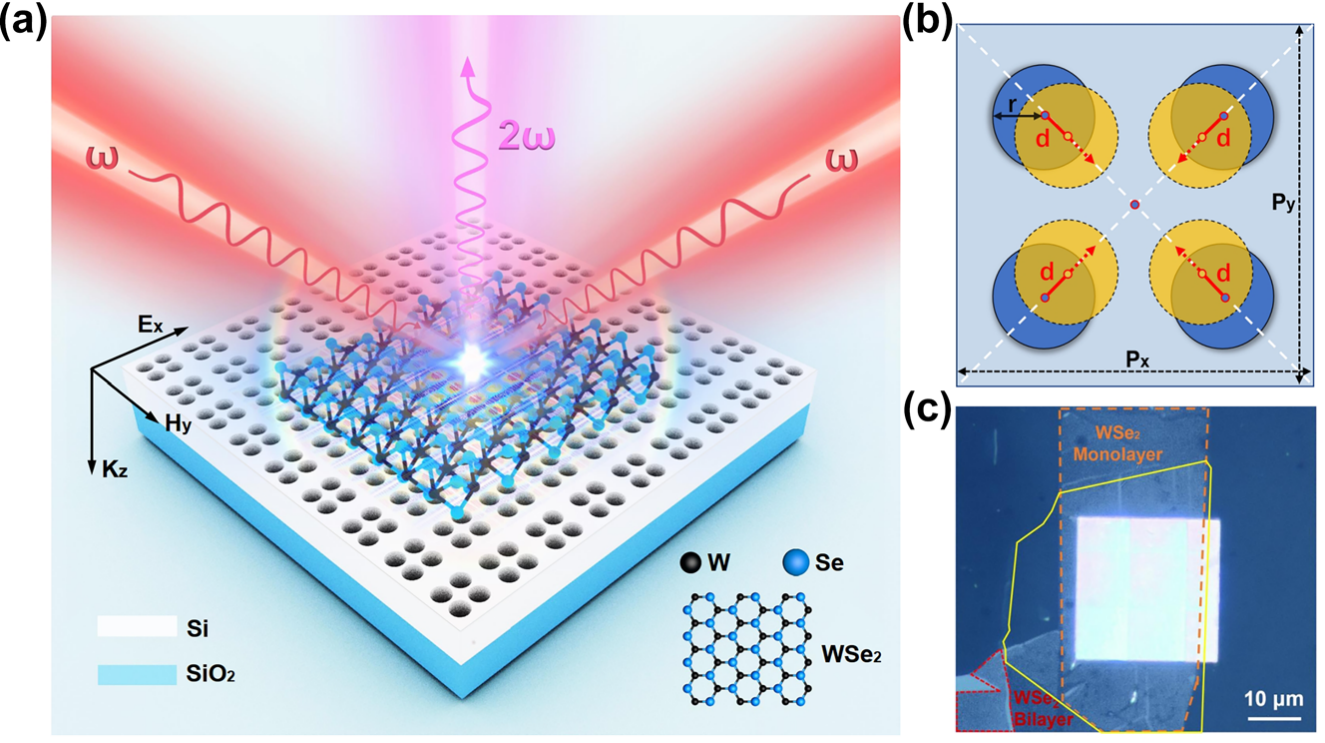
Hybrid photonic structure for enhancing SHG of 2D material. (a) Schematic of SHG from a WSe_2_ monolayer placed on top of the multiple-hole Si-metasurface fabricated on a quartz substrate. (b) Design of the unit cell with the definition of the adjustable parameter *d*, fixedly, the lattice period of metasurface *Px* = *Py* = 330 nm, the radius of the hole *r* = 50 nm. We define *d* to be 0 when the centers of the four holes are at the midpoint of half of the diagonal lines in the unit cell. When the four circular holes move diagonally toward the center of the unit cell, *d* > 0, and vice versa. (c) Optical microscope image of the fabricated device, where the orange dashed line, yellow solid line, and red dashed line show the profile of WSe_2_ monolayer, hBN and WSe_2_ bilayer, respectively. A part of WSe_2_ monolayer and hBN are outside of the metasurface, as reference.

To achieve the enhancement of SHG in 2D materials, we construct a hybrid photonic structure as shown in Figure 1. This structure comprises an all-dielectric metasurface designed on a quartz substrate, which supports a quasi-BICs mode with easy tuning of the resonant frequency, *Q*-factor, and state density. To be compatible with mature semiconductor technology, we chose silicon for the metasurface fabrication and adopted a completely flat porous structure to facilitate integration with 2D materials. The selection of silicon in our study is based on its well-established fabrication technology, whose centrosymmetric atomic structure inherently prevents intrinsic SHG from the material itself [28]. The metasurface is constructed by periodically arranging unit cells, each consisting of four equally spaced circular hole meta-atoms with the radius of 50 nm and the thickness of 250 nm. Figure 1(b) provides a schematic of the unit cell. The lattice period of the metasurface is *Px* = *Py* = 330 nm. A tunable parameter, denoted as *d*, represents the shift distance of the four holes to the center of the unit cell. The nonlinear medium, a WSe_2_ monolayer, is transferred to the top of the metasurface to construct our hybrid photonic device, as shown in the optical microscopy image in Figure 1(c). The WSe_2_ monolayer covered with an ultra-thin hexagonal boron nitride (hBN) is prepared by mechanical exfoliation from bulk crystal and transferred onto the metasurface by dry transfer method. Here, the role of the hBN layer is to protect the WSe_2_ monolayer during the transformation process. The optical micrograph in Figure 1(c) reveals minimal contrast between the bare WSe_2_ monolayer and the hBN-covered monolayer, indicating the ultra-thinness of the hBN employed. The WSe_2_ on the metasurface displays a continuous film without any fracture, indicating a clean and flat surface of the fabricated metasurface. To provide a reference for SHG without enhancement by quasi-BICs, a part of the same WSe_2_ monolayer is deliberately transferred onto the bare substrate. For such a structure, the quasi-BICs resonance could provide a strong localized light field in the 2D WSe_2_ monolayer to enhance the efficiency of second-order nonlinear processes.

**Figure 2:**
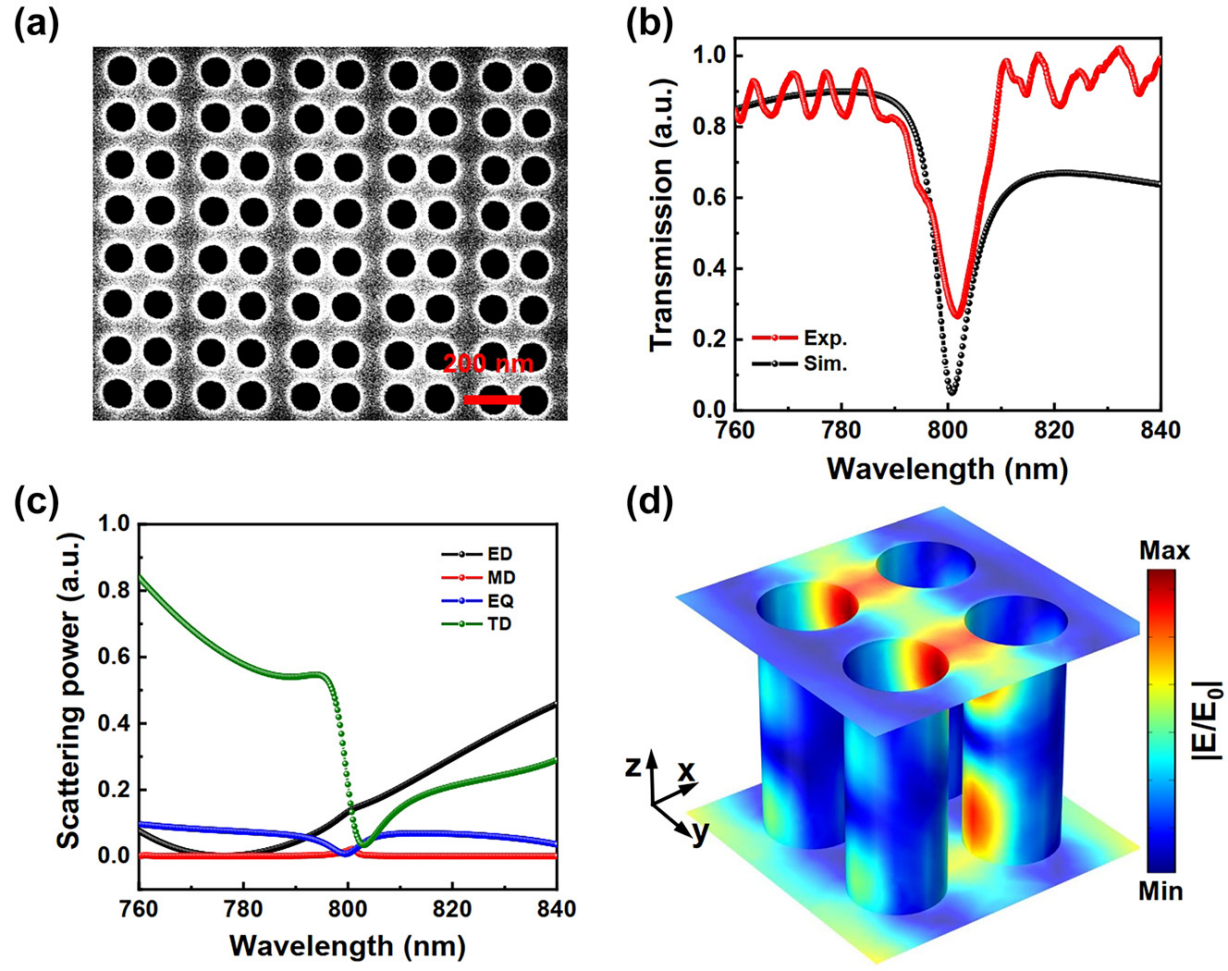
Experimental observation and theoretical analysis of the quasi-BICs mode in one of our fabricated metasurfaces. (a) Top view SEM image of the fabricated structure. The parameter *d* in this structure is about 15 nm. (b) Transmission spectra of the metasurface obtained by experiment (red-dotted line) and theoretical simulation (black-dotted line) under perpendicular incidence. (c) The normalized Cartesian multipole decomposition results in the resonance dip. (Black and red-dotted lines represent electric dipole and magnetic dipole, respectively; the blue and green dotted lines represent electric quadrupole and toroidal dipole.) (d) Simulated electric field distribution of the resonant mode in a unit cell.

The SEM image in Figure 2(a) shows one of the fabricated Si-metasurfaces with *d* equal to approximately 15 nm, which is the same as the one in Figure 1(c). Firstly, the transmission spectrum of the prepared bare metasurface in Figure 2(a) is characterized by a home-built angular-resolved spectroscopy setup under the incident of the *y*-direction-polarized (defined as TM-polarization) halogen light source. The transmission spectrum at the 0° angle of the angular-resolved spectrum is shown with the red line in Figure 2(b), which shows a strong resonance mode near 802 nm. This result is in good agreement with the numerical simulation as the black line shown in Figure 2(b). Therein, the nature of the oscillations in transmission measurements should result from the interference of the thin film caused by the substrate of the sample. As shown in Figure 1(a), our metasurface is fabricated on a quartz substrate with a thickness of approximately 1 mm, which leads to weak oscillation in the transmission measurements.

To investigate resonant mode around 802 nm, we calculate the scattered power of electric dipole (ED), magnetic dipole (MD), electric quadrupole (EQ), and toroidal dipole (TD) moments of the metasurface with *d* = 15 nm by utilizing the Cartesian multipole decomposition based on the induced current density [29]. The oscillating charge current distribution inside the nanostructure, induced by an incident linearly polarized plane wave, is expanded into a series of electric, magnetic, and toroid multipole moments (detailed information available in the methods). As the four holes move toward the center of the unit cell, the ED and TD moments can be effectively excited by external pumping, which dominates the electric field distribution around 802 nm, as illustrated by the simulation results in Figure 2(c). The interplay between these two moments finally leads to the pronounced resonance mode (dip) in the transmission spectrum in Figure 2(b). In contrast, the contribution of the EQ and MD moments to the field distribution can be ignored. The corresponding near-field distribution of this mode in the unit cell is shown in Figure 2(d), in which a strong light field hosts near the surface. Experimentally, one can employ such a resonant mode to enhance the light–matter interaction, such as boosting the SHG emission from a 2D material integrated onto the metasurface.

**Figure 3:**
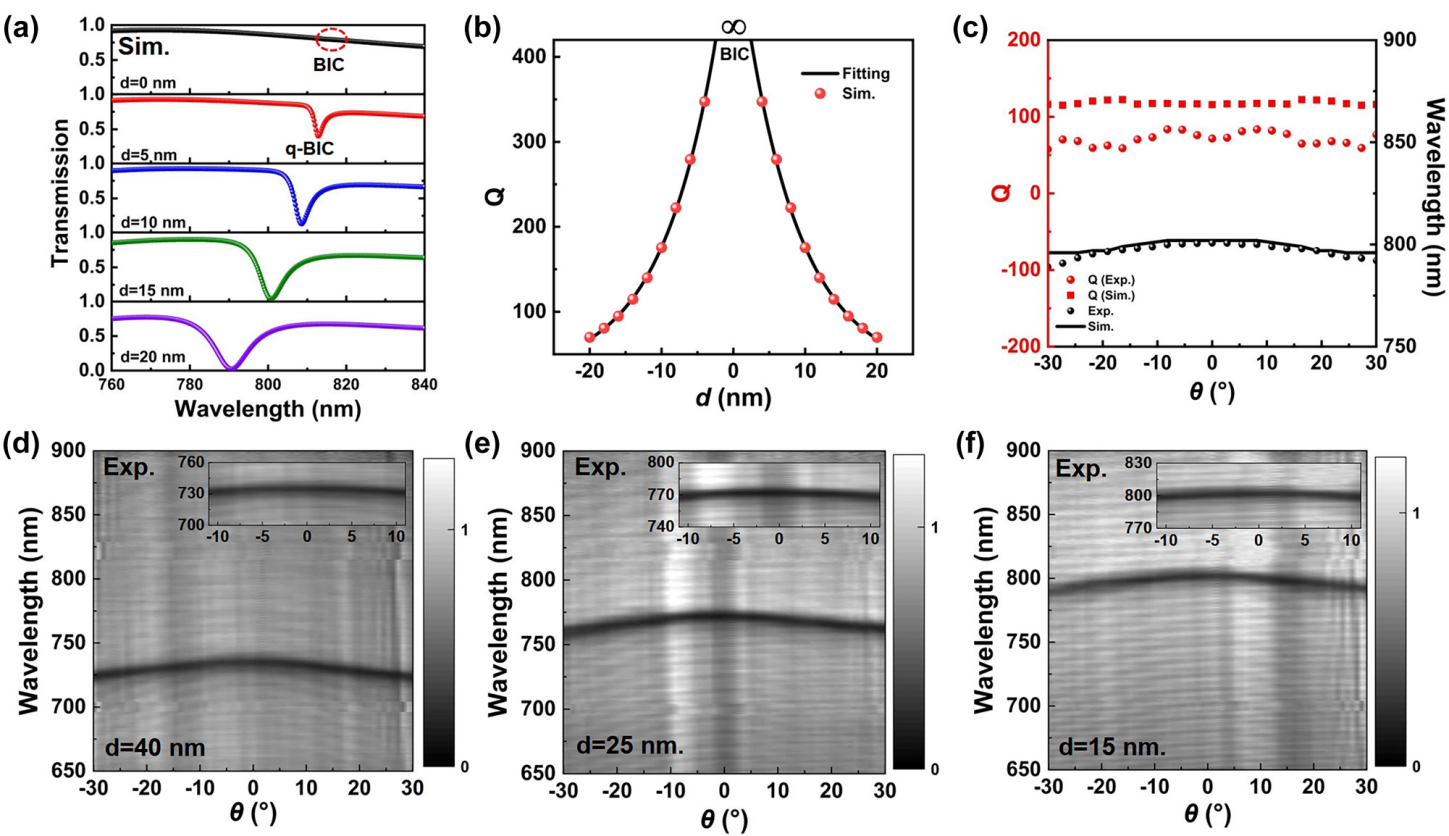
Dynamic modulation of quasi-BICs modes of the proposed metasurface. (a) Evolution of the transmission spectrum of the metasurface under different parameters *d*. (b) Dependence of the *Q* factors on the parameter *d*. The dots are the simulation result, which are fitted by the inverse quadratic law (black lines). (c) Experimental and numerical simulation of the angle-resolved transmissivity spectrum and *Q* factors of the metasurface. (d–f) Experimental angle-resolved transmissivity spectrum with different geometrical parameters.

To give an insight into the physical mechanism of the observed resonant mode in Figure 2(b), we engineer the metasurface by simultaneously shifting the four holes of the unit cell towards the center of the unit cell with a distance *d* (marked in Figure 1(b)), while keeping the hole size unchanged. For different *d*, the transmittances of metasurfaces are shown in Figure 3(a), which are obtained by numerical simulations of the linear response of the metasurface using a commercial solver based on the finite element method (see Section 4). The resonant feature disappears for *d* = 0, which indicates a symmetry-protected BIC could form around 812 nm. Since the ideal BIC mode is characterized by the absence of energy radiation outward, it exhibits a vanishing resonance with an infinite *Q* factor in the transmission spectrum, as shown in the top panel of Figure 3(a). When the conditions for BIC are slightly altered, either by breaking the in-plane symmetry or by destroying the condition of interfering resonances, BIC transitions into quasi-BIC, exhibiting finite *Q*-factors. Figure 3(b) presents an analysis of the extracted *Q* factors of these resonant modes. The *Q*-factor increases quadratically as *d* decreases, which follows an inverse quadratic law concerning [20].

In experiments, we fabricated three metasurfaces with *d* = 40 nm, 25 nm, and 15 nm, respectively. The TM-polarized angle-resolved transmission spectra of the corresponding metasurface are displayed in Figure 3(d)–(f). Notably, the quasi-BICs modes from three different metasurfaces manifest quasi-flat bands characterized by minimal dispersion at a large range of incident angles, and these modes are an almost flat band in the range from −10° to 10° (as the inserts shown in Figure 3(d)–(f)). In contrast to the optical mode with large dispersion in other cavity structures, such as plasmonic cavities [30], distributed Bragg-reflector cavities [31], and photonic crystals [32], such modes can provide a very high density of photon states for light–matter interactions. To compare with the numerical simulation, we extract the wavelengths and corresponding *Q* values of the quasi-flat band from the experimental results in Figure 3(f). As shown in Figure 3(c), the experimental measurements on wavelengths align closely with the theoretical simulation. The *Q* factors are limited by the absorption loss of silicon material and the radiative loss of the metasurface structure, but they remain unaffected by the in-plane wavevector in our detection range. Here, the in-plane wavevector (*k*_in –plane_) can be induced from the relationship: *k*_in –plane_ = 2*π* sin*θ*/*λ*, where the *θ* and *λ* are the angle and wavelength in the angle-resolved spectra, respectively. The wavevector-independent *Q* factor indicates a robust resonant mode that is not limited by the incident angle. The only discrepancy is that the *Q* value obtained in the experiment is lower than that obtained in the simulation. This is mainly because of the inevitable roughness induced in the sample preparation, and the imperfect hole prepared in metasurface.

To demonstrate the enhancement of the quasi-BICs to the light–matter interaction, we conducted room temperature measurements of the SHG responses of the hybrid metasurface-WSe_2_ structure in Figure 1(c). The metasurface (*d* = 15 nm) chosen for this experiment is shown in Figure 2(a) and has a resonant wavelength of 802 nm. Hence, we employed a femtosecond Ti: sapphire laser with a repetition rate of 80 MHz and a pulse width of 120 fs, tuned to the resonant wavelength of 802 nm. The incident laser with TM polarization is focused onto the sample using an objective with a numerical aperture of 0.5, and the beam size is around 3 μm. The SHG emission was then collected by the spectrometer after passing through a band-pass filter.

**Figure 4:**
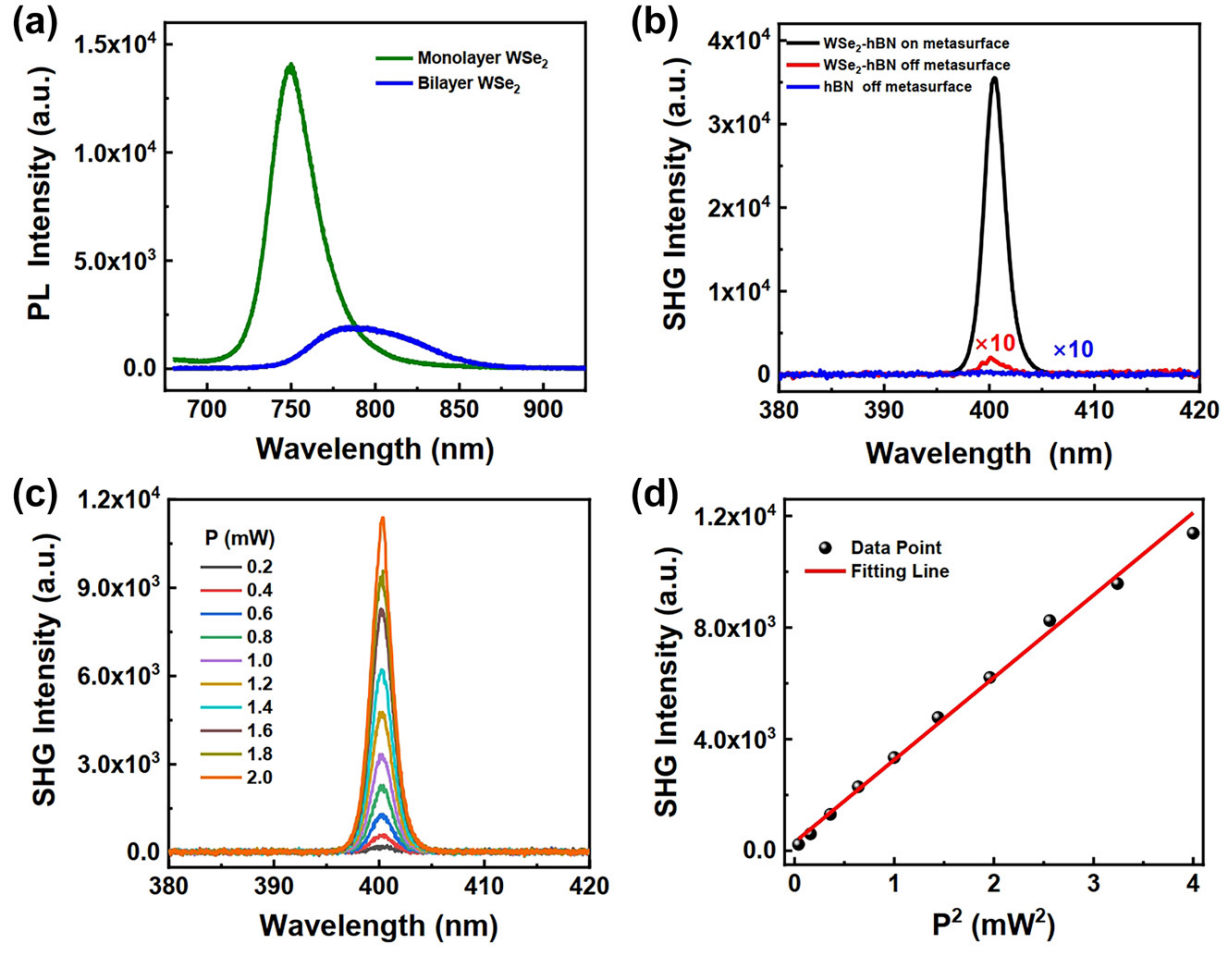
Characterization of SHG response of the hybrid metasurface-WSe_2_ structure. (a) PL spectra of the WSe_2_ flakes in different regions of the structure. (b) Measured spectra of the SHG from WSe_2_ monolayer on the metasurface with *d* = 15 nm (black) and on the bare quartz substrate (red), and the SHG from the bare hBN off metasurface (blue). The red and blue lines are magnified 10 times for visibility. (c) Power-dependent SHG studies. (d) Measured SHG intensities as a function of pumping power squared. The red line is a linearly fitting result.

The quality and the layer number of the WSe_2_ flakes are verified with the corresponding photoluminescence (PL) spectra (Figure 4(a)). The PL of the WSe_2_ located on the surface of the metasurface at room temperature under excitation of 523 nm laser is shown with the green curve in Figure 4(a). The emission peak of the direct A exciton at the wavelength of approximately 750 nm unequivocally demonstrates the monolayer structure of the WSe_2_. In contrast, the PL of bilayer WSe_2_, as shown with the blue curve, reveals an indirect excitonic emission peak at lower energy, accompanied by at least a one-order-of-magnitude reduction in the intensity of the A exciton [33].

To accurately extract the enhancement, we conduct the same measurements under identical conditions on the same monolayer WSe_2_, where one part of the WSe_2_ is on the metasurface, and the other is on a bare quartz substrate. The measurements presented in Figure 4(b) reveal that the SHG of WSe_2_ on the metasurface (black line) is enhanced up to more than 170 times relative to the sample on the bare substrate (red line). Here, the weak SHG signal (red line) detected from the WSe_2_ monolayer on the bare substrate is magnified 10 times for visibility. Under the resonant condition, the quasi-BIC provides a highly localized electric field in the 2D material, significantly enhancing the light–matter interactions and further boosting the nonlinear response. Although the *Q* factor of the observed quasi-BIC is not very high, and the absorption losses of Si and WSe_2_ are high at the wavelength of SHG emission, a dramatic SHG enhancement is still achieved in our work. In order to eliminate the effect of thin hBN on the SHG of WSe_2_ monolayer, we characterized the SHG of the bare hBN off the metasurface under the same pumping condition. As shown by the blue curve in Figure 4(b), there is no SHG emission from the hBN. The power-dependent SHG response of the metasurface-WSe_2_ structure is represented in Figure 4(c), with the SHG emission peaks centered at 401 nm. Figure 4(d) illustrates the relationship between SHG intensities and the pumping power squared. This shows explicit quadratic behavior as the pumping power increases from 0.2 to 2 mW, indicating the SHG regime before saturation. Throughout our power-dependent measurements, the emission intensity remained stable, indicating that the sample did not experience any laser-induced structural damage.

## 3 Conclusions

In this work, a tunable all-dielectric Si-metasurface hosting BIC is presented and further integrated with a WSe_2_-monolayer to enhance the SHG in the WSe_2_. By manipulating the parameters of the presented metasurface, a quasi-BICs mode with a flat band is achieved, which is confirmed experimentally by the angle-resolved spectroscopy. Benefitting from the high state density of the quasi-flat band of the quasi-BICs coupled to the TMDCs, the SHG emission experiences a sharp enhancement of more than two orders of magnitude relative to the other regions of the same sample not located on the metasurface. Our work develops a versatile and compact platform for complementing and even outperforming the capabilities of previously designed structures in both controlling optical modes and integration with 2D materials for enhancing the light–matter interactions such as boosting the efficiency of nonlinear processes.

## 4 Methods

### 4.1 Numerical simulations

Numerical simulations presented in the paper were performed using a commercial solver based on the finite element method (COMSOL Multiphysics). The simulation domain consisted of periodic multiple-hole Si-metasurfaces. Periodic boundary conditions are employed for the four lateral boundaries in the *x* and *y* directions to mimic an infinite photonic crystal. Port boundary conditions were used in the top and bottom boundaries to excite the system and to collect outgoing waves. A perfect matching layer (PML) is established above the metamaterial structure along the *z*-axis to eliminate the non-physical reflection at the boundary. The material parameters used for Si and SiO_2_ were those experimentally measured by ellipsometry in the structure used.

To analyze the electromagnetic properties of the periodic multiple-hole Si-metasurfaces. We utilize multipole expansions in the Cartesian coordinates for currents within the nanoparticle. Generally, the oscillating charge current distribution in nanoarrays, induced by the polarized plane wave of the incident ray can be expressed as a series of electric, magnetic, and multiple polar moments. The scattered power of electric dipole (ED), magnetic dipole (MD), electric quadrupole (EQ), and toroidal dipole (TD) moments can be calculated using the following formulas by employing the long-wavelength approximation [29]:

(1) $$P=\frac{1}{i\omega }\int J\left(r\right){\text{d}}^{3}r$$

(2) $$M=\frac{1}{2c}\int r\times J\left(r\right){\text{d}}^{3}r$$

(3) $$Q=3\int \text{d}r^{\prime }\left[r^{\prime }P\left(r^{\prime }\right)+P\left(r^{\prime }\right)r^{\prime }\right]$$

(4) $$T=\frac{1}{10c}\int \left\{\left(r\cdot J\left(r\right)\right)r-2\left(r\cdot r\right)J\left(r\right)\right\}{\text{d}}^{3}r$$

where *r* is the position vector, *c* is the light velocity, and *ω* is the frequency. *J*(*r*) indicates the charge current in the structure. The scattered power of each dipole, I_ED_, I_MD_, and I_TD_ can be obtained by the following formulas:

(5) $$I_{\text{ED}}=\frac{2\omega^{4}}{3c^{3}}{\left|P\right|}^{2}$$

(6) $$I_{\text{MD}}=\frac{2\omega^{4}}{3c^{3}}{\left|M\right|}^{2}$$

(7) $$I_{\text{EQ}}=\frac{\omega^{6}}{160\pi \varepsilon_{0}c^{5}}{\left|Q\right|}^{2}$$

(8) $$I_{\text{TD}}=\frac{2\omega^{6}}{3c^{5}}{\left|T\right|}^{2}$$

As shown in Figure 2(c), the scattering power reveals that the leading contribution comes solely from the electric dipole (ED) and toroidal dipole (TD) moments, consistent with the behavior of the corresponding electric field distribution.

### 4.2 Sample fabrication

The Si-metasurface on the quartz substrate is prepared by high-precision electron beam lithography (EBL). Firstly, spin coat hydrogen peroxysilane onto the substrate surface, then expose the target pattern using EBL. After development, using inductively coupled plasma dry etching to remove the unprotected silicon, completing the preparation of the array.

### 4.3 Optical measurements

Transmission spectra in our research are measured using a home-built angle-resolved spectroscopy setup. The metasurfaces are placed on the confocal plane of two objectives, i.e., the focus objective and the collection objective, which are aligned precisely on their optic axis. The incident light, a halogen lamp, is focused onto the sample by a focus objective with numerical aperture of 0.75, and the transmission light through the sample is collected by a collection objective with numerical aperture of 0.5. Therefore, the range of the angle-resolved spectra in Figure 3(d)–(f) is determined by the collection objective, and it is from −30° to +30°. In the measurements of transmission spectra, the diameter of the light beam focused on the sample is around 10 μm. The Fourier plane at the position of the back focal plane of the collection objective is imaged onto the slit of a Princeton Instruments spectrometer with a cooled charge-coupled camera. For the SHG measurements, an objective with numerical aperture of 0.5 is used for both focusing and collection.
